# Highlighting the new consensus guidelines for managing people at risk of, and with early‐stage type 1 diabetes—Relevance to clinical care in the UK

**DOI:** 10.1111/dme.15508

**Published:** 2024-12-29

**Authors:** Parth Narendran, Philip Newland‐Jones, Naresh Kanumilli, Rose Stewart, Fiona Regan, Tabitha Randell

**Affiliations:** ^1^ Diabetes Medicine, College of Medicine and Health University of Birmingham Birmingham UK; ^2^ The Queen Elizabeth Hospital Birmingham UK; ^3^ Diabetes & Endocrinology University Hospitals Southampton Southampton UK; ^4^ Manchester University Foundation Trust and Clinical Network Lead for Diabetes‐Greater Manchester Manchester UK; ^5^ Betsi Cadwaladr University Health Board Bangor UK; ^6^ Paediatric Endocrinology and Diabetes Evelina London Children's Healthcare London UK; ^7^ Paediatric Endocrinology and Diabetes Nottingham University Hospital Nottingham UK

Type one diabetes (T1D) can be identified before the need for insulin through the measurement of islet autoantibodies.^1^ The presence of a single islet autoantibody indicates risk whereas two or more, even in the absence of hyperglycaemia, is sufficient to define early‐stage T1D.^2^ T1D now can therefore be classified into the early‐stage ‘presymptomatic’ and later insulin requiring ‘symptomatic’ stages. Since autoantibodies can appear years before symptomatic presentation and the need for insulin, individuals identified with presymptomatic T1D will need education, support and monitoring leading up to insulin initiation. The recent consensus statement initiated by Breakthrough T1D (formerly the Juvenile Diabetes Research Foundation) with endorsement from other societies (see original publication) provides some guidance on how we should do so.^3^ The aim of this commentary is to highlight this article and explore how it best applies to clinical care in the UK.

Diagnosing T1D early has advantages; it reduces presentation in diabetic ketoacidosis,^4^, ^5^ reduces glycaemic exposure prior to diagnosis and facilitates glucose control in the years that follow,^6^ and provides time to prepare for a life with insulin and the associated reduction in anxiety.^7^ Furthermore, the availability of immunotherapy to delay T1D (in the USA and potentially soon in the UK^8^) is increasing interest in screening programmes to identify patients suitable for clinical intervention. Research screening programmes are now established internationally,^4^ through the ELSA (Early Surveillance for Autoimmune diabetes, www.elsadiabetes.nhs.uk) and T1DRA (Type 1 Diabetes Risk in Adults, www.t1dra.bristol.ac.uk) in the UK, and through a nationally sanctioned screening programme in Italy.^9^ Over and above this, many of us have patients identified early through clinical care or through reclassification of adults with dysglycaemia originally thought to be type 2 diabetes. The management of these patients has not previously followed any formal structure, and these consensus guidelines are therefore welcome.

There are four key areas.

First, guidance is provided around the frequency and intensity of follow‐up for single Ab as well as multiple Ab individuals, the different stages of early‐stage presymptomatic T1D, in adults and children. The pros and cons of the different approaches to glucose monitoring (glycated haemoglobin, continuous glucose monitoring, self monitoring of blood glucose) are outlined and the utility of these different approaches at different stages is discussed. Importantly, advice is also provided around safety. The importance of regular education about symptoms of diabetes and diabetic ketoacidosis is highlighted and all health professionals involved in monitoring and care of these individuals have a responsibility to provide this education. At this time there are no formal recommendations of how appropriate monitoring and follow‐up pathways may be practically implemented given this is likely to be a novel service, and this will require attention and careful consideration.

Second, the guidance highlights the urgent need to educate our health care professional colleagues around the single antibody and early stages of T1D so that they are aware of them and how to manage and support them. The roles of national professional bodies, the Association of British Clinical Diabetologists, the British Society for Paediatric Endocrinology and Diabetes, the Primary Care Diabetes Society, Breakthrough T1D and Diabetes UK will be critical here and educational webinars, written material and conference workshops are planned. Clinical coding for early‐stage T1D is now available through SNOMED (Systematized Nomenclature of Medicine Clinical Terms) as well as an ICD 10 (International classification of diabetes, 10th revision) code. Our primary care colleagues should be encouraged to implement them into their clinical interfaces to help alert to the risk of impending hyperglycaemia in patients presenting with other symptoms. Such approaches will reduce the risk of missed diagnosis and fatalities, and support with identification of cohorts for future potential pharmacological interventions. The support of the wider diabetes multidisciplinary team including nurses, dieticians, psychologists and their respective societies is essential. Whilst the guidelines suggest that there is a need for primary care to take on some of the early‐stage monitoring and managing of antibody positive children and adults, we recommend that within the UK NHS system, that these patients best sit in secondary care. This will facilitate the use of appropriate glucose monitoring systems, careful timing of insulin initiation and will also protect our busy primary care colleagues from what is an emerging T1D subspecialty. Meanwhile for our secondary care colleagues, the application of the diagnostic term ‘T1D’ to the early T1D stages allows them to use all the technology and services that we currently use for our patients already on insulin. Modelling the potential impact of screening on the expansion of clinical services suggests an initial increase but care will be less intensive than for insulin treated patients.^10^

Third, the possible need for psychosocial support is emphasised. The anxieties of living with risk of a future chronic condition have been previously outlined^11^; and while early detection and management may bring many benefits, these benefits can be reduced or in some cases outweighed if the result causes significant distress, has a negative impact on parenting and/or relationships, or triggers unhelpful coping strategies such as adoption of disordered eating behaviours or pursuit of potentially untested and unsafe ‘cures’. Patients and parents with pre‐existing difficulties with anxiety and or/depression who may be more vulnerable to experiencing difficulties adapting to and living with the knowledge of future illness may need extra support. Similarly, appropriate referral for clinical psychology for assessment and support will be an important part of the care we provide for these patients; which has significant implications for workforce given the difficulties already encountered across services in meeting the needs of people with established diabetes.

Fourth, the guidance highlights the need for further research, particularly in adults where the natural history of T1D not well understood. All interested people with early T1D should be offered trial participation. In the UK, we have the UK T1D Consortia with a clinical trial finder and the latest information in T1D research. We are also due to have a registry for all children and adults with one or more islet autoantibodies—one role is to be able contact individuals about suitable trials.

We have previously been aware of people with early‐stage T1D before the need for insulin. Many of them have historically been identified and followed up through research studies, but as a recognition of these stages and as screening programmes increase, we need to be adept at managing them in clinical care. We believe these guidelines will facilitate equitable care across the different geographical and demographic population of the UK.
